# Impact of ethnicity on the relevance of the Alcohol Use Disorders Identification Test (AUDIT) in Nepal

**DOI:** 10.1192/bji.2024.44

**Published:** 2025-05

**Authors:** Susmita Pandey

**Affiliations:** Public Health Research Department, Sakshyam Research Institute, Kathmandu, Nepal

**Keywords:** Alcohol Use Disorders Identification Test, AUDIT, alcohol use disorders, ethnicity, Nepal

## Abstract

The Alcohol Use Disorders Identification Test (AUDIT) is a ten-item standardised questionnaire to assess an individual's vulnerability to alcohol use disorders. Three of these ten questions are related to alcohol consumption. Nepal has a distinct alcohol culture, where alcohol use is socially and religiously acceptable in some caste/ethnic groups and is prohibited in others, thereby influencing the scores of AUDIT questions related to all three conceptual domains. Identifying and endorsing subsets of AUDIT questions relevant to different ethnic groups could be the way forward for effective screening of alcohol use disorders in Nepal.

The Alcohol Use Disorders Identification Test (AUDIT) is a screening tool to identify individuals who are at risk for alcohol use disorders (AUDs). Developed by the World Health Organization (WHO), the AUDIT is reported to provide an accurate measure of risk across gender, age and cultures.^1^ The AUDIT consists of ten questions formulated under three conceptual domains: hazardous alcohol use (AUDIT-C: questions 1–3), alcohol dependence symptoms (AUDIT-dependence: questions 4–Q6) and harmful alcohol use (AUDIT-harm: questions 7–10). These questions measure the experience of using alcohol within the past year. Each question from 1 to 8 has four possible responses, with scores ranging from 0 to 4. Questions 9 and 10 have three potential responses, with scores of 0, 2 and 4. Hence, the total score ranges from 0 to 40, with a higher score indicating greater AUD vulnerability.

Many epidemiological and clinical studies worldwide have used the AUDIT to screen for AUD vulnerability. It has been adapted and validated in many countries, including Nepal, and has demonstrated good psychometric properties.^2,3^ The validation study in Nepal has, however, not considered the ethnicity of the individuals. Nevertheless, globally, across different cultural groups, the validity of the AUDIT is well established, and there remains little, if any, doubt in the ability of the AUDIT to identify individuals with AUD vulnerability in different ethnic groups in Nepal. At the same time, given the ambivalent alcohol culture in Nepal, it may not be wise to completely dismiss the impact that ethnicity could have on the AUDIT score. Therefore, this opinion article has been put forward.

In Nepal, the use of alcohol is embedded in the culture of some caste/ethnic groups. Be it a significant event or incident in their life such as marriage, childbirth, death, festivities or a part of the day-to-day routine, alcohol is an inseparable part of living for almost all age groups. The culture is so deeply rooted that women during pregnancy and postpartum are given home-brewed alcohol,^4^ on the grounds of health benefits during pregnancy and to produce and sustain milk supply during postpartum. However, there are some other caste/ethnic groups where the use of alcohol is considered a sinful act against their cultural belief system. To sum up, among more than 100 caste/ethnic groups in Nepal, alcohol use is socially and culturally acceptable and even glorified in some groups (the traditionally alcohol-using caste/ethnic groups such as Janajati, Newars and Dalit), and in other groups, alcohol use is socially prohibited and considered polluting on religious grounds (the traditionally non-alcohol-using caste/ethnic groups such as Brahmins and Chettris).^5^ Nevertheless, these sociocultural disparities in alcohol consumption patterns among the traditionally alcohol-using and non-using groups are decreasing, with a rise in cross-cultural tolerance and acceptance over the past few decades. However, the very fact that alcohol use was a part of the culture for ages in some caste/ethnic groups and is just a few generations old in other caste/ethnic groups may have different implications for their AUD vulnerability.

For instance, studies show that neuroadaptation in response to alcohol use may have a hereditary component, indicating that the same amount of alcohol used by individuals with and without a family history of AUD elicits different neural responses.^6^ Schuckit et al compared people with and without a family history of AUD, and found that those with a positive family history adapt more rapidly to the presence of alcohol by showing a faster decrease in the level of prolactin following a high-dose alcohol challenge.^7^ In the case of the AUDIT, this finding may imply that the same score on the AUDIT-C may suggest different AUD vulnerability in those with and without a history of alcohol use in their family.

Moreover, it may be important to consider the individual scores of questions in the AUDIT when screening for people at risk for AUD within specific caste/ethnic groups, such as the traditionally alcohol-using groups in Nepal. For example, within this group, the scores for the first three questions (AUDIT-C) may be significantly higher in terms of the frequency and amount of alcohol consumption, potentially affecting the overall score. Apparently, a study in Nepalese adults showed that ethnicity was related to early transitions in alcohol use (opportunity, lifetime use to regular use), but not to the later transitions from regular use to the development of AUD.^8^ They found this difference when the traditionally alcohol-using caste/ethnic groups (Janajatis, Dalits and Newars) were compared with the traditionally non-alcohol-using caste/ethnic groups (Brahmins and Chettris). From this finding, we can conclude that the AUDIT-C questions could be ethnicity-dependent, whereas the AUDIT-dependence and AUDIT-harm questions are less affected by the respondent's ethnicity. This may indicate that the AUDIT-dependence and AUDIT-harm questions could be more apt in screening AUD vulnerability among the traditionally alcohol-using caste/ethnic groups, and scores on the AUDIT-C questions could be of minimal importance.

Even among the AUDIT-dependence and AUDIT-harm questions, some questions may be more valid than others in eliciting AUD vulnerability in the traditionally alcohol-using caste/ethnic groups. For instance, question 6 of the AUDIT-dependence asks if a person needs a drink in the morning to get going. For some communities within the traditionally alcohol-using caste/ethnic groups, consuming alcohol as the first drink in the morning is a regular practice, similar to consuming tea or coffee as part of their breakfast.^9^ Therefore, the framing of this question could be easily misunderstood, with respondents focusing only on the first drink aspect and potentially overlooking the aspect of needing a drink to get going after heavy drinking, which could affect the question's validity.

Also, question 10 of the AUDIT-harm, regarding a relative or friend or a doctor or another health worker being concerned about one's drinking or suggesting to cut down, could be affected by factors other than just drinking. For instance, in a traditionally alcohol-using family where almost everyone is drinking, it is less likely that another family member is concerned or would comment on the drinking of the other family member. When talking about suggestions from doctors and healthcare workers suggesting to cut down or showing concern, the accessibility of healthcare workers to specific caste/ethnic groups could be another influencing factor. Looking at the geographic distribution of the traditionally alcohol-using caste/ethnic groups in Nepal, they are more likely to be living in the remotest parts of Nepal,^10^ where accessibility to healthcare workers is minimal,^5^ thereby reducing their possibility of a suggestion from a healthcare worker on their drinking. However, because of the increased migration rate from remote areas to more urban settings, and some communities within the traditionally alcohol-using caste/ethnic groups originating from the more urban parts of the country (for instance, the Newar community in Kathmandu Valley), this explanation may not be exhaustive. Nevertheless, alcohol-related medical help-seeking is significantly higher among the traditionally non-alcohol-using caste/ethnic groups^11^ despite substantially more alcohol consumption in the traditionally alcohol-using community.^8^ Therefore, a score of ‘0’ or an answer of ‘No’ to question 10, which affects the overall score by 10%, is influenced by multiple factors rather than just alcohol use.

So, for the traditionally alcohol-using caste/ethnic groups of Nepal, the score on question 4 (impaired control over drinking) and question 5 (increased salience of drinking) of the AUDIT-dependence, and question 7 (guilt after drinking) and question 8 (blackouts) of the AUDIT-harm domain could be of major importance, and could relate directly to their AUD vulnerability when compared with the rest of the questions on the AUDIT.

Identifying a subset of questions that is more relevant to a specific group/culture/context from the standard set of questions has another advantage. Some studies choose to use a subset of a questionnaire rather than the entire questionnaire, particularly when conducting large-scale screenings or when dealing with numerous variables. In such cases, the study would benefit from using a subset of questions that relates more to the population under study, rather than using random subsets of the original questionnaire. For example, in the case of using the AUDIT in traditionally alcohol-using caste/ethnic groups of Nepal, rather than using the AUDIT-C, a subset comprising questions 4, 5, 7 and 8 could be more effective in identifying AUD vulnerability.

All in all, given the ambivalent alcohol culture in Nepal from the outset, the same score on each AUDIT question may mean different things for the traditionally alcohol-using and non-using ethnic/caste groups, in terms of AUD vulnerability. Therefore, the key to the optimal adaptation of standard questionnaires like the AUDIT could be looking deeper into the culture and context and determining what works for whom in subgroups of the population, rather than simply adapting at a country level.
